# Preliminary development of a questionnaire assessing the impact of psoriasis and psoriatic arthritis on patient's perception of sexuality

**DOI:** 10.1097/MD.0000000000012807

**Published:** 2018-10-19

**Authors:** Eric Esteve, François Maccari, Dominique Delavierre, Catherine Vicariot, Bénédicte Charles, Marc Marty, Eric Lespessailles

**Affiliations:** a Department of Dermatology, Regional Hospital of Orleans; b I3MTO Laboratory, University of Orleans, Orleans; c Hospital Begin, Saint Mandé; d Department Urology-Andrology, Regional Hospital of Orleans, Orleans; e Multidisciplinary Healthcare House, Meung sur Loire; f France Psoriasis, Paris; g Department of Rheumatology, Henri Mondor Hospital, Creteil; h Department of Rheumatology, Regional Hospital of Orleans, Orleans, France.

**Keywords:** psoriasis, psoriatic arthritis, quality of life, questionnaire, sexuality

## Abstract

Psoriasis and psoriatic arthritis impact sexuality and intimate relationships in both men and women, and can be associated with sexual dysfunctions.

The aim of this study was to develop and validate a specific questionnaire assessing the impact of psoriasis and psoriatic arthritis on patients’ sexuality. Two focus groups of patients, concerned by sexuality, were conducted in February 2015. Based on the verbatim transcripts, a content analysis was performed by a psychologist trained in qualitative procedures.

After analysis of the verbatim reports by the research group, a preliminary questionnaire comprising 22 questions was drawn up. The areas covered by the questions concerned: the quality of daily life, tolerance of the cutaneous state by the patient, tiredness, mobility and flexibility of the joints, outside activities involving movement of all or part of the body. In the following step the questions were then submitted to a panel of experts for selection using a Delphi method. The experts were questioned about item relevance and content. After expert consensus had been reached, the instructions to participants completing the questionnaire, the wording of items, and the possible answers were finalized.

The final questionnaire (comprising 14 questions) is entitled “Questionnaire of sexual quality of life perceived by patients with cutaneous and/or articular psoriasis” (short denomination: Qualipsosex). The quantitative step has not yet been conducted. This step will aim to evaluate the metrologic qualities (reliability, validity, and responsiveness) of the questionnaire and will need to be validated according to the consensus-based standards for the selection of health measurement instrument checklist.

## Abstract 2

Supplemental Digital Content is available in the text

## 1 Introduction

Psoriasis (Ps) is a chronic inflammatory disorder of the skin. Its prevalence in Caucasian populations varies from 0.9% to 8.5%.^[1]^ This immunologically mediated skin disease is associated with depressive illness, cardiovascular disease, hypertension, diabetes mellitus, obesity, and psoriatic arthritis (PsA).^[2,3]^ Recent evidence suggests that 15% to 30% of patients with Ps develop PsA.^[4]^ Furthermore 29% of patients with Ps attending dermatology clinics have undiagnosed PsA.^[5]^ PsA affects men and women equally, and is present in 8% to 10% in patients with Ps.^[6]^

Quality of life is significantly altered in patients with Ps.^[7,8]^ Beyond the skin, psoriasis negatively impacts most daily activities through the numerous comorbid conditions^[9,10]^ which are associated with this disabling disease. PsA is a complex and heterogeneous disease associated with a number of musculoskeletal abnormalities including various clinical and radiographic phenotypes with spondylitis, arthritis, enthesitis, and dactylitis.^[6]^ Thus, beyond the symptoms associated with their Ps, patients with PsA have disabling pains that negatively affect most activities when using their hands, standing for long periods of time, walking, etc.^[11,12]^

It is well established that Ps, in both men and women, is associated with sexual dysfunctions.^[13–15]^ Although sexual problems have been mostly identified and recognized in people with rheumatoid arthritis,^[16,17]^ it has been reported that other rheumatic diseases, including PsA, impact sexuality, and intimate relationships.^[18,19]^ Furthermore, it has been demonstrated that the presence of PsA was associated with a higher prevalence of altered sexuality in patients with Ps.^[14,20]^

**Figure 1:**
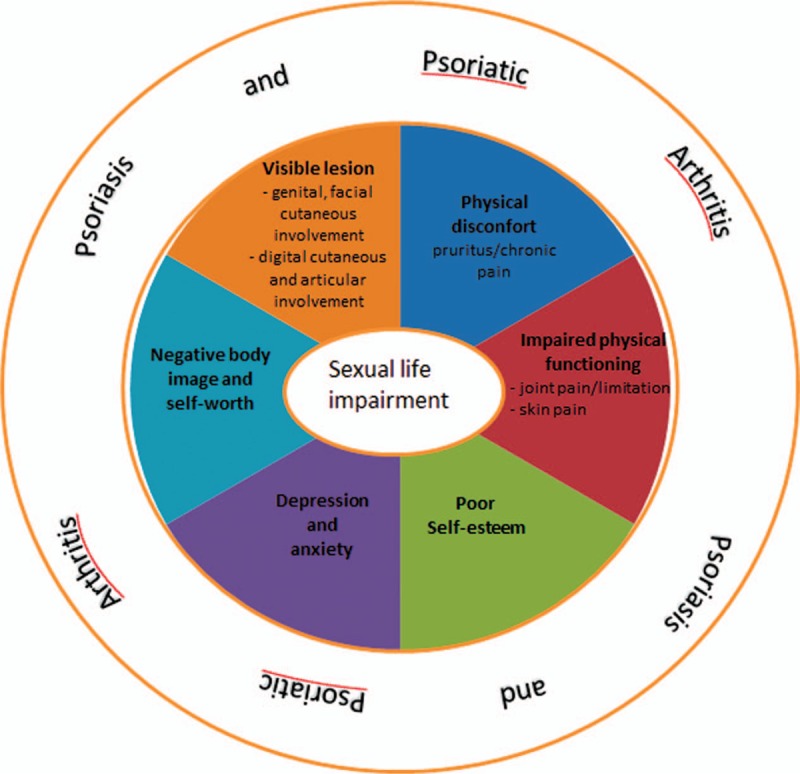
Determinants of the impact of psoriasis and psoriatic arthritis on patients’ sexuality.

However, this impact on a patient's sexuality (Fig. 1) is not currently assessed by physicians in clinical routine; only 1 question out of 10 in the most widely validated QOL questionnaire (Dermatology Life Quality Index) considers the effects of Ps on sexuality.^[21]^ Furthermore, it appears that the assessment tools available to identify the impact of Ps on sexuality are not satisfactory since the questionnaires used to determine the prevalence of sexuality problems in these patients give results that may vary from 35.5% to 71.3%.^[20]^

The aim of the present study was to develop and validate a specific questionnaire assessing the impact of Ps and PsA on patients’ sexuality.

## 2 Materials and methods

### 2.1 Qualitative step

#### 2.1.1 Defining the concept

A multidisciplinary research group met in Orleans, France, in December 2013 to define the purpose of this work and the methodology of the study. This group consisted of 2 rheumatologists (including 1 with expertise in developing and validating health questionnaires), 2 dermatologists, 1 andrologist-sexologist, 1 gynecologist, 1 member of the France Psoriasis patients’ association and 1 psychologist.

After a review of the literature in the main electronic databases including the Cochrane library, Medline, and Embase, between 1999 and 2015, the main themes were extracted by the research group to create an interviewer guide to conduct semistructured interviews. A qualitative study based on focus group interview methods was conducted.^[22]^

The present study was reviewed and approved by the research ethics board of the Centre Hospitalier Regional d’Orleans (March 9, 2017, no: 2017/15-ID RCB: 2017-A00283-50).

### 2.2 Recruitment and participants

Outpatient members of France Psoriasis (the most representative patients’ association on Ps in France) were invited by advertisement to participate in focus group interviews if they had been diagnosed as having Ps, PsA, or both. In addition, patients had to be concerned by issues of sexual health and intimacy.

To ensure response diversity, a minimum of 3 and a maximum of 10 participants were required.

### 2.3 Focus group design

Two focus groups of patients (members of France Psoriasis), concerned by sexuality, were conducted and moderated in February 2015 by a female psychologist trained in this type of interview. All the patients were volunteers. The focus group of men was performed separately from that of women. Each focus group lasted 1 to 2 hours.

The preamble of the discussion concerned the impact of PS and PsA on intimacy and patient's motivations for discussing this topic. Neither therapies nor relations with health professionals were discussed.

### 2.4 Content analysis and item generation

Based on the verbatim transcripts, a content analysis was performed by a psychologist trained in qualitative procedures. This content analysis was then further discussed by the research group including first a specialist in qualitative thematic content analysis methods, and second a patient participating in the research group to check the final concepts for face validity.

### 2.5 Item selection

The items generated in the previous step were then submitted to a panel of experts for selection using a Delphi method.^[23]^ The experts were questioned about item relevance and content. Finally, after reaching expert consensus, instructions to participants completing the questionnaire, wording of items and possible answers were finalized.

### 2.6 Determination of the relevance and potential ceiling or floor effects of the factorial structure of the questionnaire

Forty patients, all members of France Psoriasis were recruited to assess the clarity (score 0 to 9), the relevance and potential ceiling or floor effects of the various items.

### 2.7 Quantitative step

This step has not yet been conducted. It will aim to evaluate the metrologic qualities (reliability, validity, and responsiveness) of the questionnaire and will need to be validated according to the Consensus-based Standards for the selection of health Measurement Instruments (COSMIN) checklist.^[24]^

## 3 Results

The focus groups consisted of 6 women and 3 men aged between 30 and 59. All patients had cutaneous psoriasis and psoriatic arthritis. Among the patients 6/9 patients had a biotherapy, and 6/9 a local treatment. All patients had had sexual intercourse at least once in the previous 6 months. After analysis of the verbatim reports by the research group, a preliminary questionnaire comprising 22 questions was drawn up.

The number of questions was reduced by the Delphi method by a panel of 12 medical doctors (4 dermatologists, 4 rheumatologists, 2 gynecologists, 2 urologists). At this stage the version comprised 14 questions with 5 possible answers for each, evaluating the impact of cutaneous psoriasis and/or cutaneo-articular psoriasis on sexuality and 3 questions on the overall sexual quality of life.

A pilot survey of 40 patients, all members of France Psoriasis, was then conducted to assess the clarity (score from 0 to 9) and the relevance of the items proposed. Of the 40 questionnaires sent, 36 were returned. No item posed a problem of clarity. Items with a “floor” or “ceiling” effect were removed as well as redundant issues.

A preamble allows the patients to indicate whether they are concerned by sexuality or not and whether they wish to answer the questionnaire or not. It contains 10 questions (rated from 0 to 4) on the sexual quality of life specific to the disease and explores the following dimensions: seduction, approach, and sexual relations. The 4 questions related to sexual quality of life as a whole are rated from 0 to 10 (numerical scale of 11 points).

The areas covered by the questions concern: the quality of daily life, tolerance of the cutaneous state by the patient, tiredness and his/her experience, mobility and flexibility of the joints, outside activities involving bodily activity and/or baring part of the skin (sport, swimming pool in particular), the partner's accompaniment and his/her attitude towards the patient.

The final questionnaire is entitled “Questionnaire of sexual quality of life perceived by patients with cutaneous and/or articular psoriasis” (short denomination: Qualipsosex) (see in the Appendix, Supplemental Content).

## 4 Discussion

Although the consequences of rheumatoid arthritis (RA) on sexuality are now recognized and properly assessed,^[25]^ the impact on sexual life in Ps and PsA patients is not yet specifically assessed. This paper aimed to present the methods and rationale for the development of a questionnaire allowing for the appraisal of the impact of Ps and PsA on the multidimensional perception of sexuality.

In this preliminary work, various issues pertaining to different fields, including dermatology, rheumatology, gynecology, urology and psychology were raised.

According to the World Health Organization, sexual health is defined by “…a state of physical, emotional, mental and social well-being in relation to sexuality; it is not merely the absence of disease, dysfunction or infirmity. Sexual health requires a positive and respectful approach to sexuality and sexual relationships, as well as the possibility of having pleasurable and safe sexual experiences, free of coercion, discrimination and violence. For sexual health to be attained and maintained, the sexual rights of all persons must be respected, protected and fulfilled.”^[26]^ The concept of sexual health goes far beyond sexual activity itself. The reduction or loss of desire, the role of fatigue, the perceived risk of interruption of sexual intercourse, the role of the partner, the specific role of the disease in relation to body image and capacity but also relational aspects may intervene in the relationship between Ps and PsA and sexuality.^[18]^ Specifically, sexual problems associated with Ps and PsA are not solely associated with organic causes; self-esteem, depression, or changes in body image may also intervene in the psychologic repercussions of these diseases. The development of such a questionnaire should contribute to a better knowledge and understanding of the many-facetted reality of sexual problems in Ps and PsA patients. In addition, it will help both health professionals and patients to communicate on sexuality,^[17]^ and to systematize the assessment of sexual health in Ps and PsA patients. These goals are directly in line with the recent French government initiative of a strategic national plan for sexual health (2017–2030), including improving the appraisal of sexual health in people affected with chronic diseases.^[27]^

A further step is needed to complete the validation of the questionnaire. The metrologic qualities (reliability, validity, and responsiveness) of the questionnaire need now to be validated according to the COSMIN checklist.^[24]^

The reliability of the questionnaire will be assessed by the following criteria:

- Internal consistency: This property tests whether the dimensions determined during the creation of the scale are consistent, that is, whether each question of each dimension is more correlated with the score of the dimension to which it belongs or only to the overall score.
- Measurement error Bland and Altman method^[28]^ and the reliability test which assess the accuracy of the questionnaire.

The validity of the questionnaire will be assessed by the following criteria:

- Content validity which tests whether the questionnaire measures what it aims to measure. It is defined by the stages of development of the questionnaire.
- Construct validity which tests correlations of the questionnaire with questionnaires exploring the same domain.
- Validation on criterion (criterion validity) which tests correlations of the questionnaire with the activity of the disease.

Evaluation of the questionnaire's responsiveness assesses the ability of the questionnaire to detect a change in the quality of sexual life perceived by the patient when his or her clinical condition changes.

An observational multicentric, prospective study with a follow-up at 3 and 6 months is currently being conducted. One hundred and fifty patients are scheduled to participate in the study. The protocol was approved on February 16, 2017 by the ethics committee CPP Est I.

## Acknowledgment

The authors thank the patients and the association (France Psoriasis) who took part in this study. The authors also acknowledge Nukleus for methodologic help in developing the wording and content of the questionnaire. The authors also thank Najat Gouyette MSD, Laurent Hertau UCB Pharma France, and Jean-Baptiste Quiniou Celgene.

## Author contributions

**Conceptualization:** Eric Esteve, François Maccari, Dominique Delavierre, Catherine Vicariot, Bénédicte Charles, Marc Marty, Eric Lespessailles.

**Data curation:** Eric Esteve, François Maccari, Dominique Delavierre, Catherine Vicariot, Bénédicte Charles, Marc Marty, Eric Lespessailles.

**Formal analysis:** Eric Esteve, François Maccari, Dominique Delavierre, Catherine Vicariot, Bénédicte Charles, Marc Marty, Eric Lespessailles.

**Investigation:** Eric Esteve, Eric Lespessailles.

**Methodology:** Eric Esteve, Eric Lespessailles.

**Supervision:** Eric Esteve, Marc Marty, Eric Lespessailles.

**Validation:** Eric Esteve, Marc Marty, Eric Lespessailles.

**Writing – original draft:** Eric Esteve, Eric Lespessailles.

**Writing – review & editing:** Eric Esteve, François Maccari, Dominique Delavierre, Catherine Vicariot, Bénédicte Charles, Marc Marty, Eric Lespessailles.

Eric Lespessailles orcid: 0000-0003-1009-8518.

## Supplementary Material

Supplemental Digital Content
