# Supplementary material for: Preliminary development of a questionnaire assessing the impact of psoriasis and psoriatic arthritis on patient's perception of sexuality
Source: Medicine (Baltimore). 2018 Oct 19;97(42):e12807. doi: 10.1097/MD.0000000000012807 (PMC6212286; doi:10.1097/MD.0000000000012807)
Supplement: Supplemental Digital Content [file medi-97-e12807-s001.doc]

Supplemental Content

Appendix. Patient-reported quality of sexual life questionnaire among patients suffering from skin psoriasis and/or psoriatic arthritis

**Patient-reported quality of sexual life questionnaire among patients suffering from skin psoriasis and/or psoriatic arthritis**

The aim of this questionnaire is to assess the impact of your skin psoriasis and/or psoriatic arthritis on your perception of the quality of your sexual life. This assessment will help improve your treatment.

Do you consider that this questionnaire concerns you? Yes No

Do you wish to answer the questionnaire? Yes No

You suffer from: Skin psoriasis

Psoriatic arthritis

Both

For each of the following questions, **circle the number** that best corresponds to your experience in the last 3 months

| **Q1** | **Do you feel that your disease makes you less attractive sexually?** | | | | | | |
| --- | --- | --- | --- | --- | --- | --- | --- |
|  | Not at all | 0 | 1 | 2 | 3 | 4 | All the time |
| **Q2** | **Do you feel that your disease diminishes your desire to seduce?** | | | | | | |
|  | Not at all | 0 | 1 | 2 | 3 | 4 | All the time |
| **Q3** | **In your opinion, does your disease diminish your sexual desire?** | | | | | | |
|  | Not at all | 0 | 1 | 2 | 3 | 4 | All the time |
| **Q4** | **Are you afraid to be touched by your partner(s)?** | | | | | | |
|  | Not at all | 0 | 1 | 2 | 3 | 4 | All the time |
| **Q5** | **In your opinion, is/are your partner(s) afraid to touch you?** | | | | | | |
|  | Not at all | 0 | 1 | 2 | 3 | 4 | All the time |
| **Q6** | **Are you afraid to touch your partner(s)?** | | | | | | |
|  | Not at all | 0 | 1 | 2 | 3 | 4 | All the time |
| **Q7** | **Do you feel self-conscious about being seen undressed before having sexual intercourse?** | | | | | | |
|  | Not at all | 0 | 1 | 2 | 3 | 4 | All the time |
| **Q8** | **Does the pain and stiffness in your back and joints prevent you from having satisfying sexual relations?** | | | | | | |
|  | Not at all | 0 | 1 | 2 | 3 | 4 | All the time |
| **Q9** | **If your sexual organs are affected, does this prevent you from having satisfying sexual relations?** | | | | | | |
|  | Not at all | 0 | 1 | 2 | 3 | 4 | All the time |
| **Q10** | **Do you feel that the tiredness linked to your disease is an obstacle to having satisfying sexual relations?** | | | | | | |
|  | Not at all | 0 | 1 | 2 | 3 | 4 | All the time |

The following 4 questions concern your overall sexual quality of life. For each question, **circle the number** that best corresponds to your experience in the last 3 months.

| **A1:** | **In the last 3 months, has your skin problem impaired the quality of your sexual life?** | | | | | | | | | | | | |
| --- | --- | --- | --- | --- | --- | --- | --- | --- | --- | --- | --- | --- | --- |
|  | Not at all | 0 | 1 | 2 | 3 | 4 | 5 | 6 | 7 | 8 | 9 | 10 | Extremely |
| **A2:** | **In the last 3 months, has your psoriatic rheumatism impaired the quality of your sexual life?** | | | | | | | | | | | | |
|  | Not at all | 0 | 1 | 2 | 3 | 4 | 5 | 6 | 7 | 8 | 9 | 10 | Extremely |
| **A3:** | **Do you expect the medical treatment of your disease to improve the quality of your sexual life?** | | | | | | | | | | | | |
|  | Not at all | 0 | 1 | 2 | 3 | 4 | 5 | 6 | 7 | 8 | 9 | 10 | Extremely |
| **A4:** | **Are you distressed by the lack of interest shown by doctors for the quality of your sexual life?** | | | | | | | | | | | | |
|  | Not at all | 0 | 1 | 2 | 3 | 4 | 5 | 6 | 7 | 8 | 9 | 10 | Extremely |
